# Charge-transfer-induced nesting antiferromagnetism in 2D hydrogen-bonded organic frameworks

**DOI:** 10.1093/nsr/nwag176

**Published:** 2026-03-19

**Authors:** Yiyang Yin, Yang Song, Lizhi Zhang, Yuyang Zhang, Shixuan Du

**Affiliations:** National Center for Nanoscience and Technology, Beijing 100190, China; University of Chinese Academy of Sciences and Institute of Physics, Chinese Academy of Sciences, Beijing 100190, China; National Center for Nanoscience and Technology, Beijing 100190, China; National Center for Nanoscience and Technology, Beijing 100190, China; University of Chinese Academy of Sciences and Institute of Physics, Chinese Academy of Sciences, Beijing 100190, China; University of Chinese Academy of Sciences and Institute of Physics, Chinese Academy of Sciences, Beijing 100190, China

**Keywords:** spin-split antiferromagnet, 2D, hydrogen-bonded organic framework

## Abstract

Non-relativistic spin-split (NRSS) antiferromagnets (AFMs) have become a research focus in the field of antiferromagnetic spintronics due to their advantages such as avoiding spin dephasing caused by strong spin–orbital coupling and having no stray magnetic fields. Γ-split AFMs are a distinct class of NRSS AFMs, featuring overall spin splitting specifically at the Brillouin zone’s Γ point. To date, their realization has been confined to a handful of inorganic materials. Molecular frameworks are a promising platform for achieving multiple exotic properties and tunability due to their diverse ligand types and symmetries. Herein, we propose that Γ-split AFMs can be realized in 2D hydrogen-bonded organic frameworks (HOFs) via nesting honeycomb and Kagome lattices. In these ‘nesting AFMs’, each sublattice is ferromagnetic with opposite spin polarization, constructed separately using donor and acceptor molecules. Density functional theory calculations confirm that five designed nesting HOFs are nesting AFMs. Importantly, the ferromagnetic ordering of each sublattice originates from charge transfer between hydrogen-bond-connected donors and acceptors. This work provides a practical strategy to design novel low-dimensional molecule-based NRSS AFMs for antiferromagnetic spintronics.

## Teaser

In this work, spin-split antiferromagnetism is realized in 2D hydrogen-bonded organic frameworks via a nesting lattice strategy, achieving spin-polarized bands through charge transfer for spintronic applications.

## INTRODUCTION

The recent discovery of spin-split antiferromagnetism (AFM), arising independently of spin–orbit coupling (SOC), has prompted a re-evaluation of the traditional definition of AFM [1–5]. In magnetic systems, time reversal symmetry (*θ*), spatial reversal symmetry (*I*), translation symmetry (*T*) and spin reversal operation (*U*) play important roles. Conventional colinear antiferromagnets (AFMs) host *UT* symmetry such that operation *U* and *T* can both convert polarized sublattices from one direction to another, ensuring spin degeneracy when SOC is absent. When *θIT* and *UT* symmetries are broken, spin degeneracy is no longer protected and spin splitting can occur, even without SOC, which is termed non-relativistic spin-split (NRSS) AFM [3,4].

**Figure 1.**
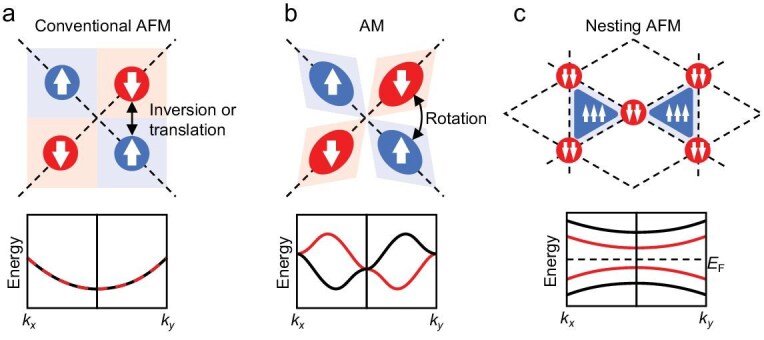
Schematics of (a) conventional AFM, (b) AM and (c) nesting AFM. Blue areas represent spin-up sites while red areas represent spin-down sites, as the white arrows point to. Black arrows represent the symmetry operators that connect the spin-up and spin-down sublattices, which are (a) inversion or translation and (b) rotation.

Altermagnetism (AM) is classified as one type of NRSS AFM. Unlike conventional AFM with symmetry protection (as shown in Fig. 1a), AM systems break *θIT* or *UT* symmetries but possess *UR* symmetry (*R* refers to a rotation operation) as shown in Fig. 1b [3,6]. Due to the existence of *UR* symmetry, altermagnets exhibit anisotropic spin-split band structures, which remain degenerate at the Γ point. If the rotation symmetry *UR* is broken as well, the band structures of altermagnets would further split at the Γ point, leading to ferromagnetic (FM)-like band configurations and preserving a zero net spin. Such magnetism is named Γ-split AFM [7], also known as fully compensated ferrimagnetism or half-metallic antiferromagnetism [8–10]. Compared to altermagnets, Γ-split AFMs exhibit fully polarized bands that can generate spin-polarized current while preserving the zero net spin of AFM. This unique property provides Γ-split AFM with distinct advantages in spintronic devices. To date, altermagnets have been reported in inorganic and organic systems [11–15]; however, Γ-split AFM materials have been confined to inorganic systems [16,17].

Theoretically, Γ-split AFM can be realized via specific strategies, such as Janus structure, staggered potential and element substitutions [10]. Most strategies focus on external tuning of magnetic states. By nesting two weakly coupled FM sublattices with different symmetries and opposite spin to break *UT* and *UR*, intrinsic Γ-split AFM can be achieved in two-dimensional (2D) monolayer materials naturally. In inorganic magnetic systems, the interaction between two sublattices is relatively strong, making it difficult to maintain the independent spin polarization distributions of two FM sublattices. However, in molecular frameworks, the naturally weak interaction between molecules makes them suitable for realizing Γ-split AFM with this strategy.

In this work, we construct 2D hydrogen-bonded organic frameworks (HOFs) using non-magnetic donor and acceptor molecules by nesting honeycomb and Kagome (HK) lattices, in which the (HK) sublattices are occupied by donors or acceptors separately. We select hexaaminobenzene (HAB) and 2,3,6,7,10,11-hexaaminotriphenylene (HATP) as donors, with 1,4-benzoquinone (BQ) and anthraquinone (AQ) as acceptors. Twelve HOFs with nesting lattices are constructed with the corresponding molecules. Density functional theory (DFT) calculations find that seven of them are thermodynamically stable. Electronic band structures reveal that five of them exhibit the Γ-split AFM state. Spatial spin distribution analysis indicates that each sublattice is FM, with the HK sublattices possessing antiparallel spin polarization. The unique magnetic configuration is accordingly named ‘nesting AFM’. Additionally, molecular dynamics simulations confirm the structural stability of the nesting-AFM HOFs on selective substrates.

## RESULTS AND DISCUSSION

### Definition of nesting AFM

The schematics in Fig. 1 illustrate the differences between conventional AFM, AM and the nesting AFM proposed in this work. For conventional AFM and AM, the spin-split sublattices are connected by certain symmetry operation (as shown in Fig. 1a and b), leading to spin degeneracy at certain high symmetry points or paths, while in nesting AFM (Fig. 1c), the symmetry between two sublattices is further broken, so that no symmetry operation can transfer one sublattice into the other. Since the polarized sublattices are not connected by any symmetry, the spin-up and spin-down bands completely split in the whole Brillouin zone. It is worth noting that after splitting, the spin-up and spin-down bands locally form an FM-like pattern, but globally show zero net magnetic moment like AFM, which is the origin of the nesting-AFM terminology.

### Design and construction of nesting-AFM HOFs

**Figure 2.**
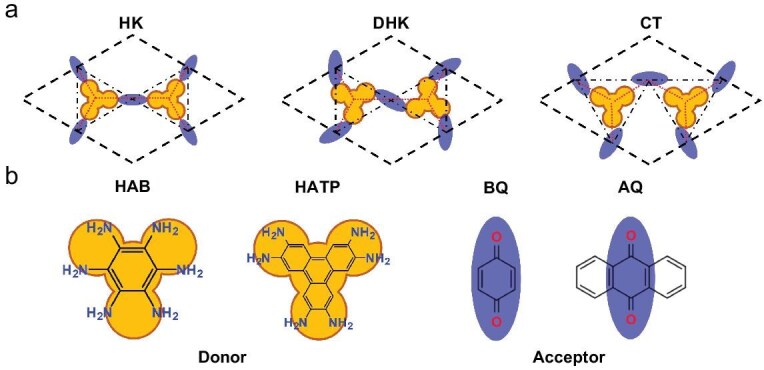
Design schematics and organic building blocks of nesting-AFM HOFs. (a) Schematics of HK, DHK and CT lattices. The black dashed line represents the unit cell, the red dotted lines represent the honeycomb sublattice, and the black chain line represents the Kagome sublattice. The yellow *C*_3_-symmetric and blue *C*_2_-symmetric symbols denote the donor and acceptor molecules, respectively. (b) Donor molecules with *C*_3_ symmetry and acceptor molecules with *C*_2_ symmetry.

Hydrogen-bonded networks are naturally suited for realizing nesting AFM since hydrogen bonds can enable charge transfer between closed-shell molecules with weak interaction between sublattices, so that the sublattices can present independent features [18]. Following this design strategy, a series of HOFs with nesting lattices formed by two types of molecules are proposed, where one type of molecule serves as donor and the other type as acceptor. Therefore, considerable charge transfer is induced by hydrogen bonds from the sublattice formed by donors to the other sublattice formed by acceptors to trigger the nesting AFM. HK and its two variant lattices considered in this work are shown in Fig. 2a. The first model is the typical HK lattice, where the donor molecules sit on the honeycomb sites and the acceptor molecules sit on the Kagome sites. The second model is the distorted HK (DHK) lattice. In this case, the molecules sit on the same sites as the HK lattice but with a different orientation. The third model is the coloring-triangle (CT) lattice, which has been proved to exhibit similar band properties to HK lattices [19–21]. All lattice models require one group of molecules to exhibit 3-fold symmetry and the other group of molecules to show 2-fold symmetry. Figure 2b shows the donor and acceptor molecules with the corresponding symmetry considered in this work. Donor molecules, namely HAB and HATP, are characterized by six amidogen groups, while acceptor molecules BQ and AQ feature two keto groups, allowing them to serve as *C*_3_ and *C*_2_ symmetry building blocks, respectively. All molecules are closed-shell structures that exhibit no intrinsic magnetic moment. The designed HOFs resemble hexagonal unit cells with the molecules connected by R–NH_2_⋅⋅⋅O=R hydrogen bonds with bond lengths of 1.55–1.75 Å.

Utilizing the donor and acceptor molecules, 12 HOFs are constructed and investigated. Their thermodynamic stability is evaluated by the formation energy *E*_form_, defined as the energy difference between the HOF and the corresponding molecules. Structures with *E*_form_ < 0 are taken as stable structures. We refer to these structures by their lattice pattern and corresponding molecules.

HOF structures are named according to their molecule compositions and lattice model. For example, an HK lattice formed by HAB molecules and BQ molecules is referred to as HAB–BQ^(HK)^. In the family of the HOFs designed, seven thermodynamically stable structures are identified. Their lattice parameters, lattice symmetry, magnetic ground states and hydrogen bond energy *E*_bond_ are presented in Table 1. Based on formation energy, distorted HK lattices and CT lattices are energetically more favorable than HK lattices in most cases, as we can see in the energy difference between HAB–BQ^(HK)^, HAB–BQ^(DHK)^ and HAB–BQ^(CT)^. This can be explained by considering that half of the hydrogen bonds feature a close-to-180° acceptor angle H⋅⋅⋅O−C [164.3° in HAB–BQ^(CT)^ and 178.4° in HAB–BQ^(DHK)^] in the distorted HK and CT lattices, while all hydrogen bonds have a 133.8° bond angle. The closer-to-180° acceptor angle leads to a closer distance between the H atom and the negative charge center of the oxygen atom, resulting in stronger bond strength. The preference of the DHK lattice and the CT lattice varies with different molecules, depending on the competition of hydrogen bonds and repulsion between hydrogen atoms of donor and acceptor molecules. The configuration with stronger repulsion where the hydrogen atoms are closer would be unfavored in terms of energy. *E*_bond_ values of the hydrogen bonds are evaluated by averaging the formation energy *E*_form_ over each bond. Depending on different molecules and lattice structures, the *E*_bond_ varies from −4.65 kcal/mol [HAB–AQ^(CT)^] to −9.77 kcal/mol [HAB–BQ^(DHK)^]. The second weakest hydrogen bond among the seven HOFs features an *E*_bond_ of −5.32 kcal/mol. In comparison, the *E*_bond_ of the hydrogen bonds between water molecules is about −4.96 to −4.93 kcal/mol, lower than that of HAB–AQ^(CT)^ but higher than that of HAB–BQ^(DHK)^. Moreover, according to our calculations, the *E*_bond_ of the hydrogen bond in experimentally reported HOF formed by tetrahydroprotoberberine (THPB) molecules [22] is −5.36 kcal/mol. Therefore, in terms of energy, the HOFs reported herein exhibit considerable stability, comparable to that of experimentally realized 2D HOFs.

**Table 1.** Lattice parameters, lattice symmetries, magnetic ground states and formation energies of the seven HOFs shown in Fig. 2. *a*_lat_ refers to the lattice constant, *E*_bond_ refers to the bonding energy of the hydrogen bond, and FiM refers to ferrimagnetic state.

	*a*_lat_ (Å)	Point group	Magnetism	*E*_bond_ (kcal/mol)
HAB–BQ^(HK)^	24.04	*D*_6h_	FiM	−5.35
HATP–BQ^(HK)^	33.52	*D*_6h_	Nesting AFM	−7.91
HAB–BQ^(DHK)^	24.22	*C*_6h_	FM	−9.77
HATP–BQ^(DHK)^	32.65	*C*_6h_	Nesting AFM	−7.67
HAB–BQ^(CT)^	20.97	*D*_3h_	Nesting AFM	−9.07
HAB–AQ^(CT)^	24.66	*D*_3h_	Nesting AFM	−4.65
HATP–BQ^(CT)^	29.57	*D*_3h_	Nesting AFM	−8.37

Figures S1–S7 present the atomic and band structures of the seven HOFs. The bands near the Fermi level present typical three-band Kagome bands contributed by acceptor molecules, and four-band honeycomb bands contributed by donor molecules, agreeing with the lattice model. The band widths of the topological band pattern are within 0.2 eV, smaller than but still comparable to those of the theoretically reported metal–organic frameworks like Cu–DCA [23]. It is worth noting that five out of seven HOFs, namely HATP–BQ^(HK)^, HATP–BQ^(DHK)^, HAB–AQ^(CT)^, HAB–BQ^(CT)^ and HATP–BQ^(CT)^, presented a nesting-AFM ground state with a zero net spin polarization, while the spin-up and spin-down bands split in an FM fashion. To further evaluate the magnetic ground state, a series of colinear AFM configurations is considered, as shown in Fig. S8. The energy comparison of different colinear magnetic configurations is shown in Table S1. For the five HOFs with nesting-AFM ground state, the energy of their nesting-AFM states is at least 36 meV per unit cell lower than the second lowest energy state [HAB-AQ^(CT)^ case]. Non-collinear circumstances are also considered and verified in HAB-AQ^(CT)^ systems, as shown in Fig. S9. The results show that the in-plane magnetic states exhibit significantly higher energy than the colinear nesting-AFM states. This could result from the fact that the polarized bands near the Fermi level are mainly contributed by *p_z_* electrons of molecules. As the result shows, nesting AFM is favored in five of the seven HOF cases.

Interestingly, all HOFs exhibiting nesting-AFM ground state show topological flat bands (TFBs) at the Fermi level, as presented in Figs S3–S7. Generally, TFBs are unique energy bands with no dispersion, resulting from destructive interference of Bloch wave functions [24,25], and have gained attention for their strong electron–electron interactions, making them ideal for exploring many-body physics phenomena [25–29]. Recently, HOFs hosting TFBs have also been theoretically predicted [30] and experimentally realized [22], indicating the potential and applicability of 2D HOFs. As partial filling of TFBs can lead to spin polarization and possible FM state [31], the origin of FM ground states of HAB–BQ^(HK)^ and HAB–BQ^(DHK)^ is clear, as they both have flat bands sitting on the Fermi level when spin is not involved. Such half-metallic properties with flat bands at the Fermi level are further confirmed by METAGGA calculations, as presented in Fig. S10.

### Electronic structures of HATP–BQ^(DHK)^ and HAB–AQ^(CT)^

**Figure 3.**
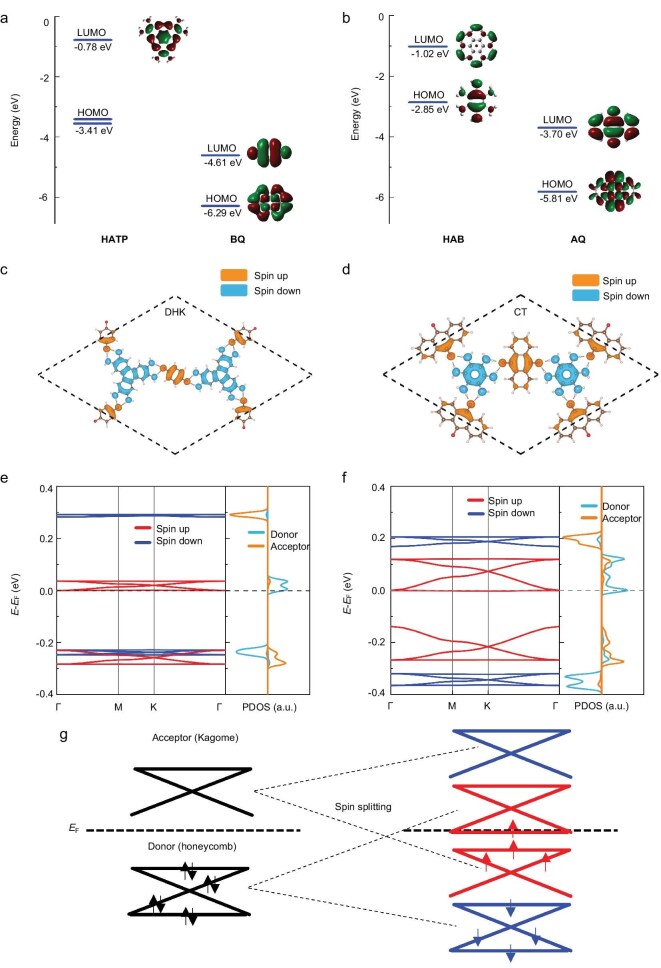
Electronic structures of HATP–BQ^(DHK)^ and HAB–AQ^(CT)^ HOFs. (a and b) DFT-calculated frontier molecular orbitals of (a) HATP and BQ, (b) HAB and AQ. Electronic structures of HATP–BQ^(DHK)^ and HAB–AQ^(CT)^ HOFs. (c and d) Spin-density distribution of HATP–BQ^(DHK)^ and HAB–AQ^(CT)^. The orange and blue represent the spin-upward and -downward polarization, respectively. (e and f) Spin-polarized band structures and PDOS projected on donor and acceptor molecules of HATP–BQ^(DHK)^ and HAB–AQ^(CT)^. (g) Schematic illustration of the electron occupation near the Fermi level in the non-magnetic and nesting-AFM states.

To evaluate the design strategy of nesting-AFM HOFs, we perform further investigation of the HOFs with the nesting-AFM state. We select the HATP-BQ^(DHK)^ and HAB-AQ^(CT)^ configurations to represent the DHK and CT lattices, respectively, and their electronic structures and spin-density distributions are illustrated in Fig. 3. As shown in the molecular frontier orbits shown in Fig. 3a and b, the lowest unoccupied molecular orbits (LUMOs) of acceptors are lower than the highest occupied molecular orbits (HOMOs) of donors, which leads to the strong charge transfer between them. Figure 3c and d demonstrate the spin-density distributions of HATP–BQ^(DHK)^ and HAB–AQ^(CT)^. Two types of molecules exhibit upward and downward spin polarization, respectively, thus forming a pair of inequivalent spin-polarized sublattices. The charge transfer is assessed through Bader charge analysis, indicating a net electron transfer of 2.4 electrons per unit cell in HATP–BQ^(DHK)^ and 2.3 electrons per unit cell in HAB-AQ^(CT)^ between the donor and acceptor sublattices. This indicates a significant charge transfer between donor and acceptor molecules. Therefore, spin polarization of each sublattice originates from the electron doping of closed-shell molecules induced by hydrogen bonds, aligned with the design strategy of nesting AFM. If acceptors are replaced with molecules that have significantly higher LUMO energy levels (such as difluorobenzene molecules), the charge transfer is weakened and the HOF will turn to non-magnetic, as shown in Fig. S11.

Figure 3e presents the band structure and projected density of states (PDOS) of HATP–BQ^(DHK)^, and Fig. 3f presents the band structure and PDOS of HAB–AQ^(CT)^. Bands near the Fermi level exhibit patterns matching the typical honeycomb four-band model and the Kagome three-band model. The PDOS indicates that the four bands mainly arise from the donor molecule contributions, while the three bands arise from the acceptor molecule contributions, in agreement with the lattice model. The Kagome bands and honeycomb bands split in opposite directions, as schematically presented in Fig. 3g. The density of states reveals that there exist some minor electron contributions from donors in the acceptor sublattice band patterns, and vice versa. This further indicates the existence of charge transfer between donor and acceptor molecules due to the hydrogen bonds, which could be the origin of different band widths of spin-polarized bands.

### Calculation of the transition temperature

**Figure 4.**
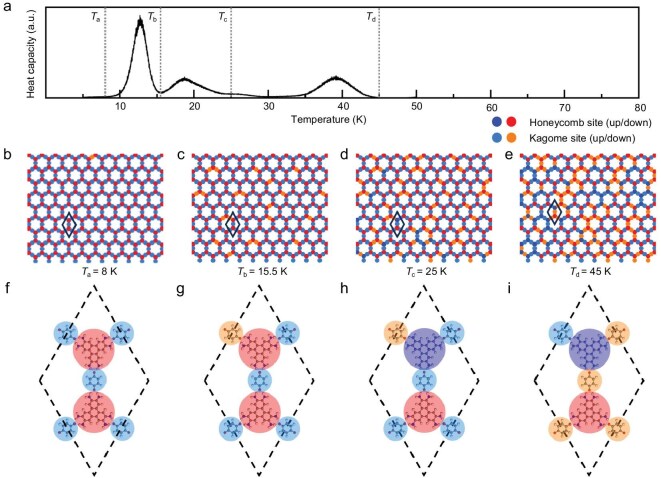
Monte Carlo simulation of nesting-AFM HK lattice with Ising model. Simulation is performed with a 200 × 200 supercell. (a) Transition temperature of nesting AFM extracted from the peak points of the heat capacity curve. (b) Spin distribution snapshot at 8 K. Nesting-AFM pattern can be seen. (c) Spin distribution snapshot at 15.5 K after the transition point. Long-range order is compromised and the Kagome sublattice shows partial disorder. (d) Spin distribution snapshot at 25 K. The Kagome sublattice shows stronger disorder and the honeycomb sublattice exhibits disorder in certain regions. (e) Spin distribution snapshot at 45 K. Spin-polarization distribution presents a random feature. (f–i) Typical magnetic configurations corresponding to (b–e).

After identifying the nesting-AFM ground state in the designed HOFs, we further investigated the magnetic phase transition behavior and spin dynamics of the nesting-AFM HK lattice using Monte Carlo simulations based on the Ising model, with the key results presented in Fig. 4. To ensure statistical reliability, the simulations were performed on a 200 × 200 supercell. Here we demonstrate the result based on the HATP–BQ^(HK)^ lattice as a representative, as the HK lattices and DHK lattices present similar behaviors. In the simulations, each molecule is treated as an individual lattice point with spin polarization.

Figure 4a depicts the transition temperature of the nesting-AFM phase, extracted from the peak positions of the heat capacity curve corresponding to distinct magnetic phase transitions. Figure 4b–e show zoom-in spin-distribution snapshots at four representative temperatures (8 K, 15.5 K, 25 K and 45 K), illustrating how spin-polarization distributions change with increasing temperature. At 8 K, the nesting-AFM state is roughly preserved. Both honeycomb and Kagome sublattices exhibit an FM arrangement, but with different polarization directions. Only several points in the Kagome sublattice show antiparallel polarization to the other Kagome lattice points due to the limitation of simulation accuracy, as presented in Fig. S12. As the temperature increases to 15.5 K, beyond the phase transition peak at 12.5 K, the magnetic phase transition occurs and the long-range order is compromised. Random spin distributions emerge in localized regions of the Kagome sublattice, while the honeycomb sublattice retains its FM ordering. At 25 K, the spin distribution shows more significant disorder in the Kagome sublattice, and disorder also starts to appear in the honeycomb sublattice. When the temperature reaches 45 K, both the honeycomb and Kagome sublattices lose spin ordering, resulting in random distribution of spin polarization across the entire 200 × 200 supercell. This simulation shows that the nesting-AFM transition temperature among the presented HOFs is relatively low, which properly results from the weak exchange interaction between molecules via hydrogen bonds.

To verify the dynamical stability of the HOFs, phonon dispersion calculations are performed on freestanding HAB–BQ^(HK)^ as a representative. Results show dynamical instability due to rotational freedom of its 2-fold-symmetric ligands in freestanding conditions (as shown in Fig. S13). However, given that such materials can stabilize on appropriate substrates, such as the Cu–DCA framework [23,32], calculations of HOFs on appropriate substrates are conducted. Considering that the hydrogen bonds are weaker than covalent bonds, and the band widths of some HOFs are quite small, large stretch should be avoided in case the band patterns become isolated band levels like individual molecules. We discovered that certain transition metal dichalcogenide materials show decent lattice mismatch with some of the HOFs presented. The electronic band structures and spin-density distributions of HATP–BQ^(CT)^ on WSe_2_ are summarized in Fig. S14. The nesting-AFM state of the HOF structure is well preserved, as is the spin-polarized flat band at the Fermi level. *Ab initio* molecular dynamics simulations are performed to verify dynamical stability of the named HOFs on the corresponding substrates, with the NVT ensemble under 300 K using the Vienna *ab initio* simulation package (VASP) with machine learning acceleration. The results show that the lattice symmetry of HATP–BQ^(DHK)^ on WSe_2_ is well preserved, as presented in Fig. S15.

## CONCLUSION

In summary, we propose a new strategy for realizing NRSS AFM and have reported a family of HOFs with HK and CT lattices following the strategy. We select HAB and HATP as donor molecules, and BQ and AQ as acceptor molecules. We have discovered seven thermodynamically stable structures and five of them host Γ-split AFM states as designed, where the honeycomb sublattice and Kagome sublattice exhibit opposite spin-polarization directions, leading to FM-like band structures with no net spin polarization. We refer to this new Γ-split AFM as nesting AFM. The exotic magnetic state originates from the charge transfer from donor molecules to acceptor molecules. Further exploration of HOFs on substrates proves the experimental practicality of these HOFs. Our findings reveal a new avenue for realizing the NRSS AFM states in the seldomly used HOF systems and provide a new strategy for designing 2D organic materials with exotic properties, such as spin-polarized flat bands.

## METHODS

All the calculations performed in this work are based on first-principles DFT calculations using the VASP [33] with the projector augmented-wave method. The Perdew–Burke–Ernzerhof generalized gradient approximation [34] is adopted for the exchange–correlation functional with D3 correction [35] to include the van der Waals interactions. The energy cutoff of the plane-wave basis sets is 400 eV, and a 3 × 3 × 1 *k*-mesh grid is used for the self-consistent total energy calculation. In all the calculations, a 15 Å vacuum layer is used, and all atoms are fully relaxed until the residual force on each atom is less than 0.01 eV/Å. The electronic structure calculation results are further examined with the METAGGA method using SCAN functional [36]. Molecular dynamics simulations are also conducted by VASP with machine learning acceleration provided by the package [37]. The Monte Carlo simulation is performed via the Ising model utilizing the Wolff algorithm.

## Supplementary Material

nwag176_Supplemental_File
