# Supplementary material for: Charge-transfer-induced nesting antiferromagnetism in 2D hydrogen-bonded organic frameworks
Source: Natl Sci Rev. 2026 Mar 19;13(7):nwag176. doi: 10.1093/nsr/nwag176 (PMC13094543; doi:10.1093/nsr/nwag176)
Supplement: nwag176_Supplemental_File [file nwag176_supplemental_file.pdf]

Special Topic: Molecular Quantum Materials

Supplementary Data for

"Charge-Transfer Induced Nesting Antiferromagnetism in Two-dimensional Hydrogen-Bonded Organic Frameworks"

Yiyang Yin[1, 2†], Yang Song[1†], Lizhi Zhang[1*], Yuyang Zhang[2], Shixuan Du[2*]

1. National Center for Nanoscience and Technology, Beijing, 100190, China
2. University of Chinese Academy of Sciences and Institute of Physics, Beijing 100190, China

*Corresponding author Email: zhanglz@nanoctr.cn, sxdu@iphy.ac.cn

†Equally contributed to this work.

Contents

1. Atomic structure and band structures of 7 thermodynamically stable HOFs under different magnetic states.

2. Schematics of colinear AFM configurations considered in this work.

3. Energy of different magnetic states of the seven HOFs.

4. Demonstration of the non-colinear magnetic configurations.

5. Verification of the electronic properties with DFT-SCAN functional.

6. Comparison of different acceptor molecule choices, with DFB molecule as representative.

7. Larger snapshot (25×30 supercell) of the Monte Carlo simulation at 8K.

8. Dynamic stability analysis with HAB-BQ$^{(HK)}$ as representative.

9. Atomic configuration, band structure and molecular dynamic simulation results of HATP-BQ$^{(CT)}$ on WSe_2.

1. Atomic structure and band structures of 7 thermodynamically stable HOFs under different magnetic states.

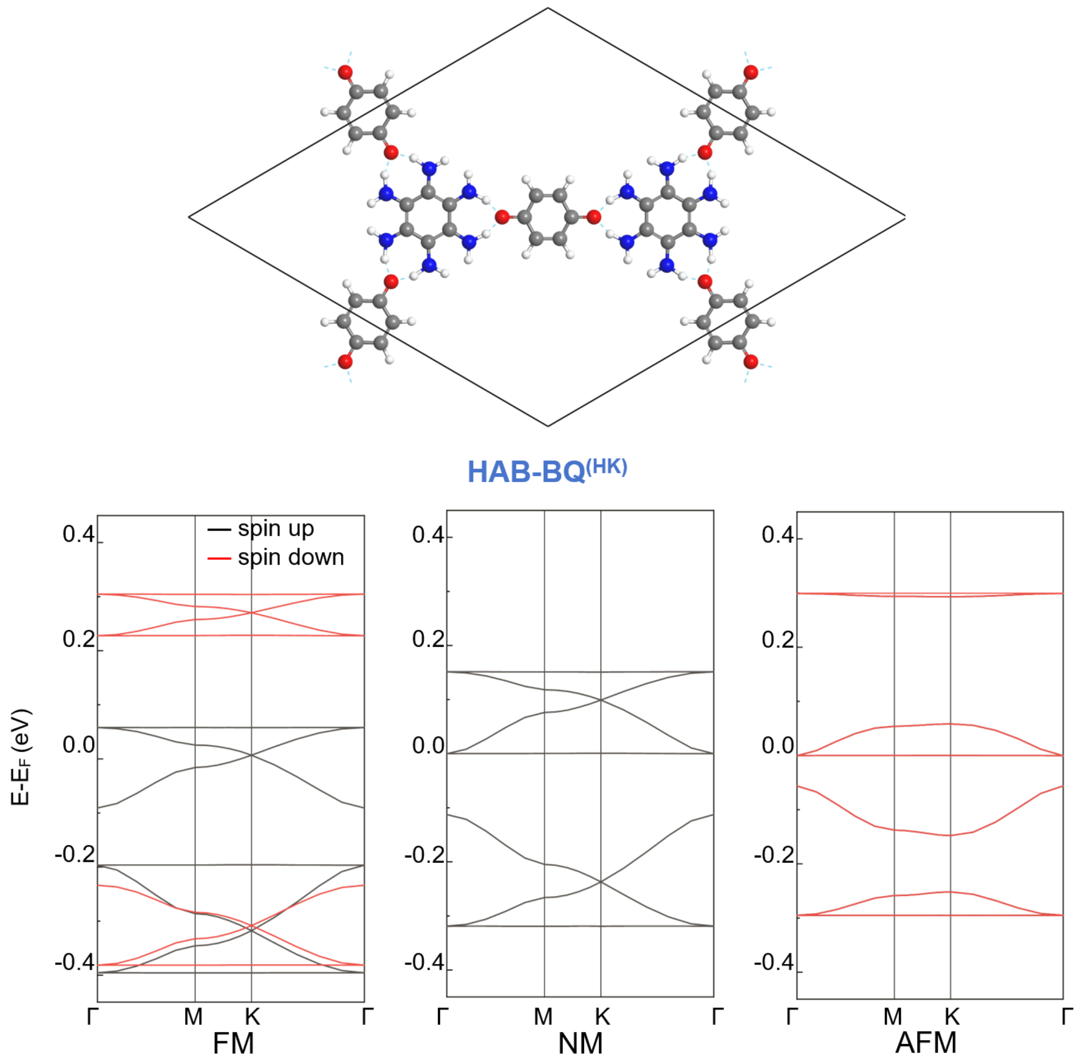

Figure S1. Atomic configuration and band structure of HAB-BQ$^{(HK)}$ HOF under different magnetic states.

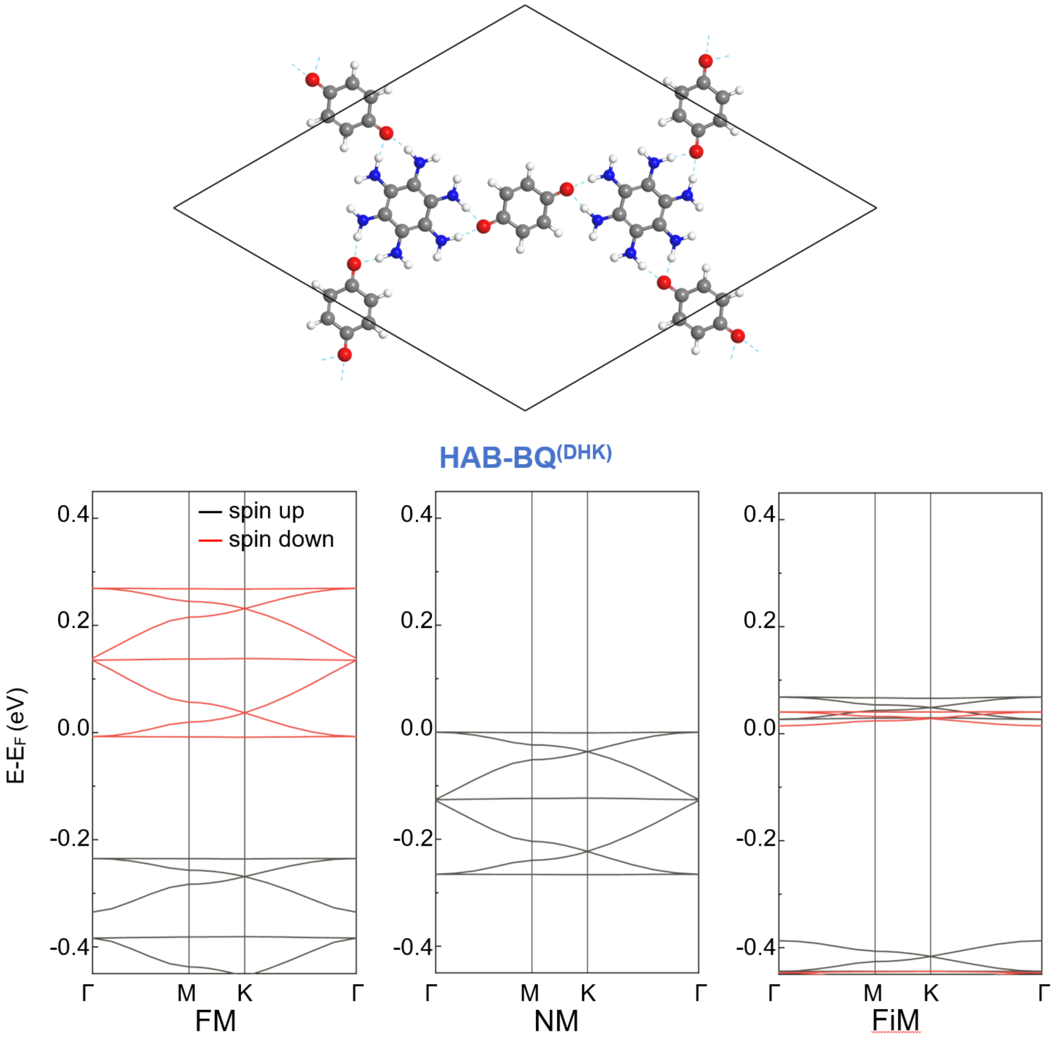

Figure S2. Atomic configuration and band structure of HAB-BQ$^{(DHK)}$ HOF under different magnetic states.

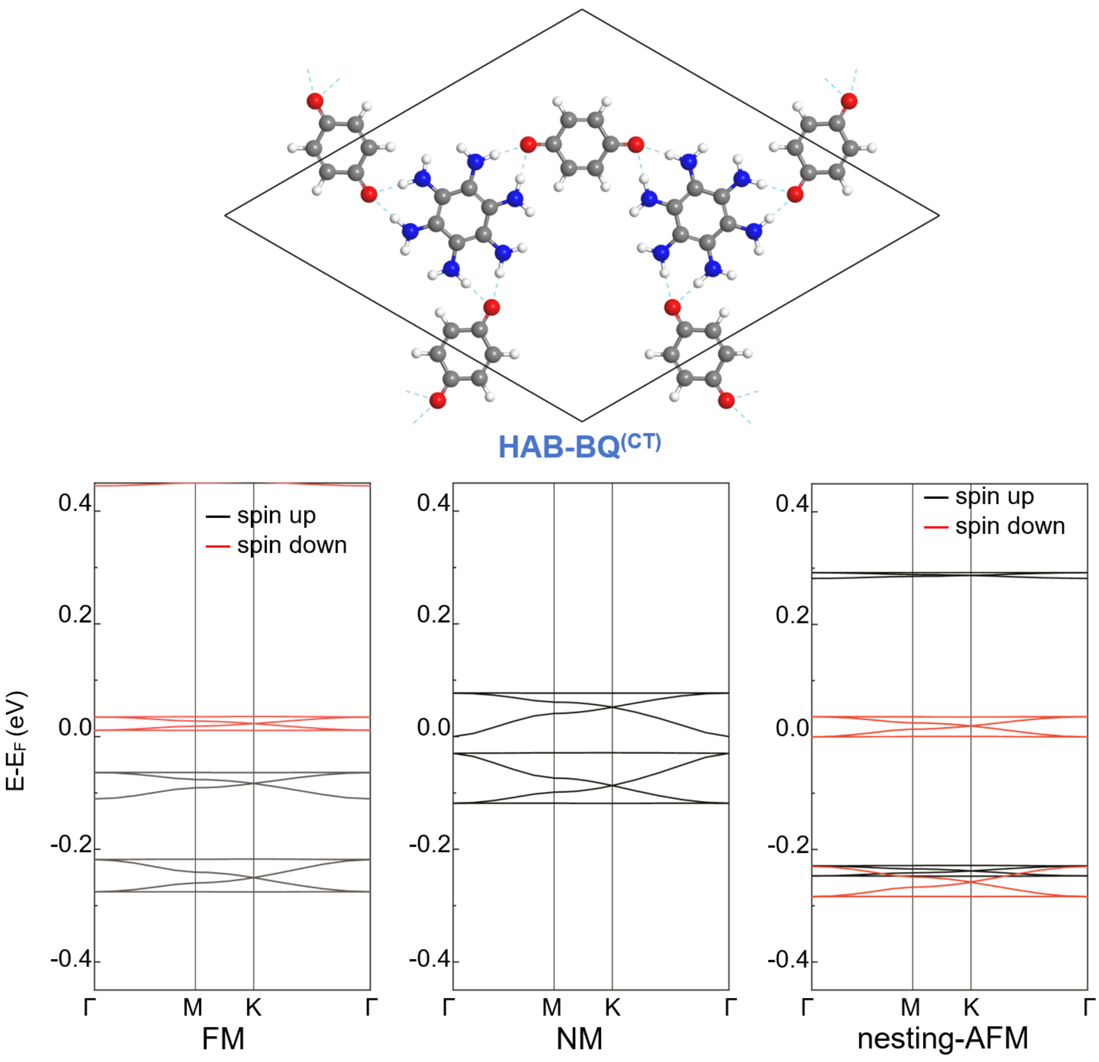

Figure S3. Atomic configuration and band structure of HAB-BQ$^{(CT)}$ HOF under different magnetic states.

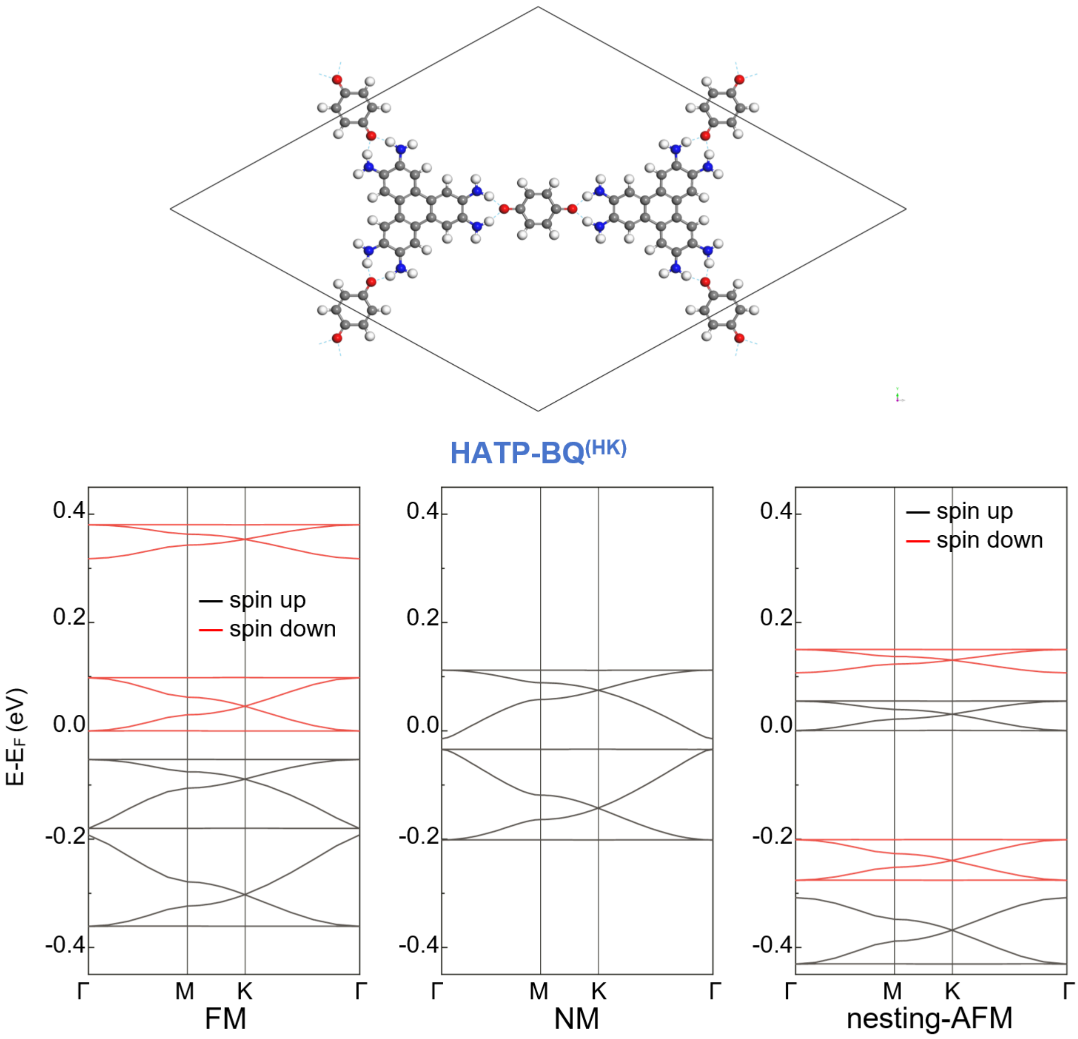

Figure S4. Atomic configuration and band structure of HATP-BQ$^{(HK)}$ HOF under different magnetic states.

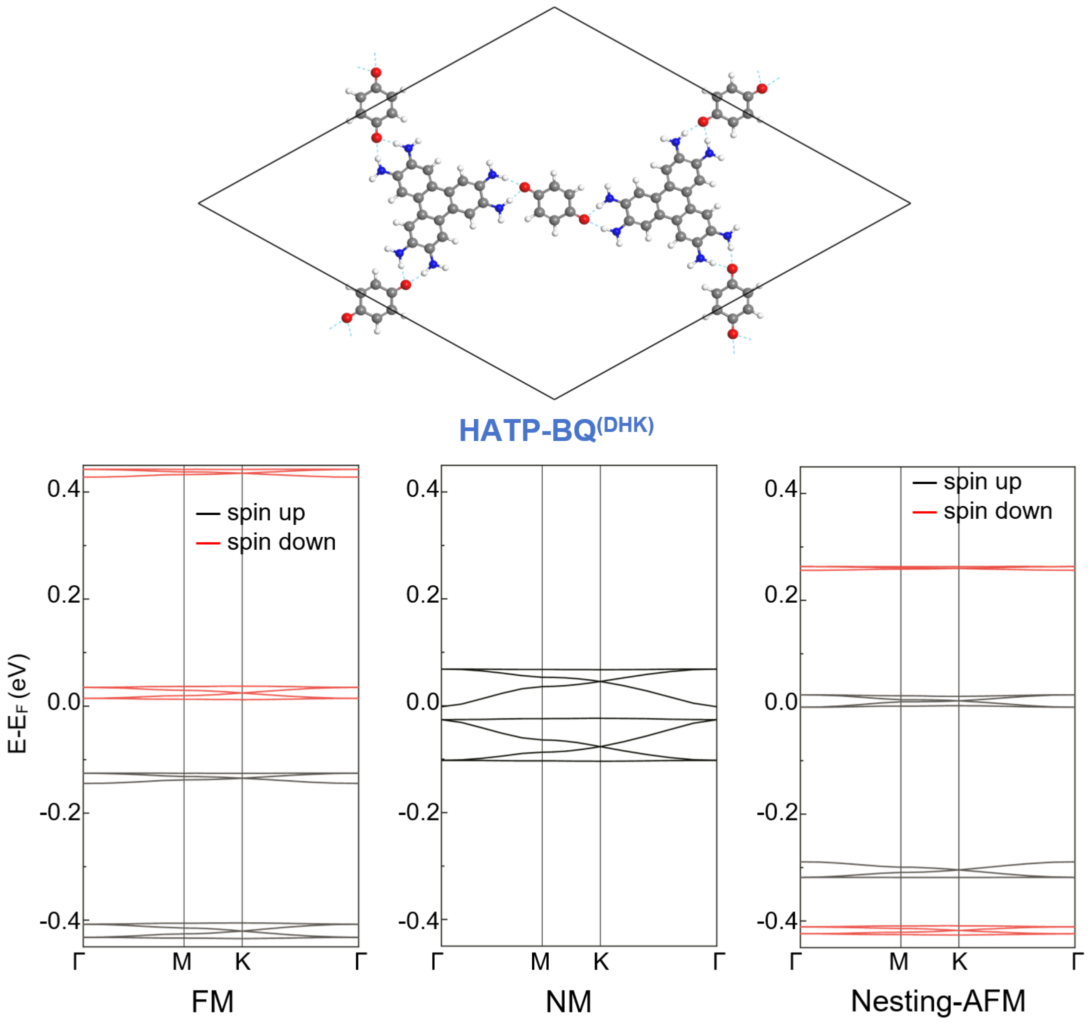

Figure S5. Atomic configuration and band structure of HATP-BQ$^{(DHK)}$ HOF under different magnetic states.

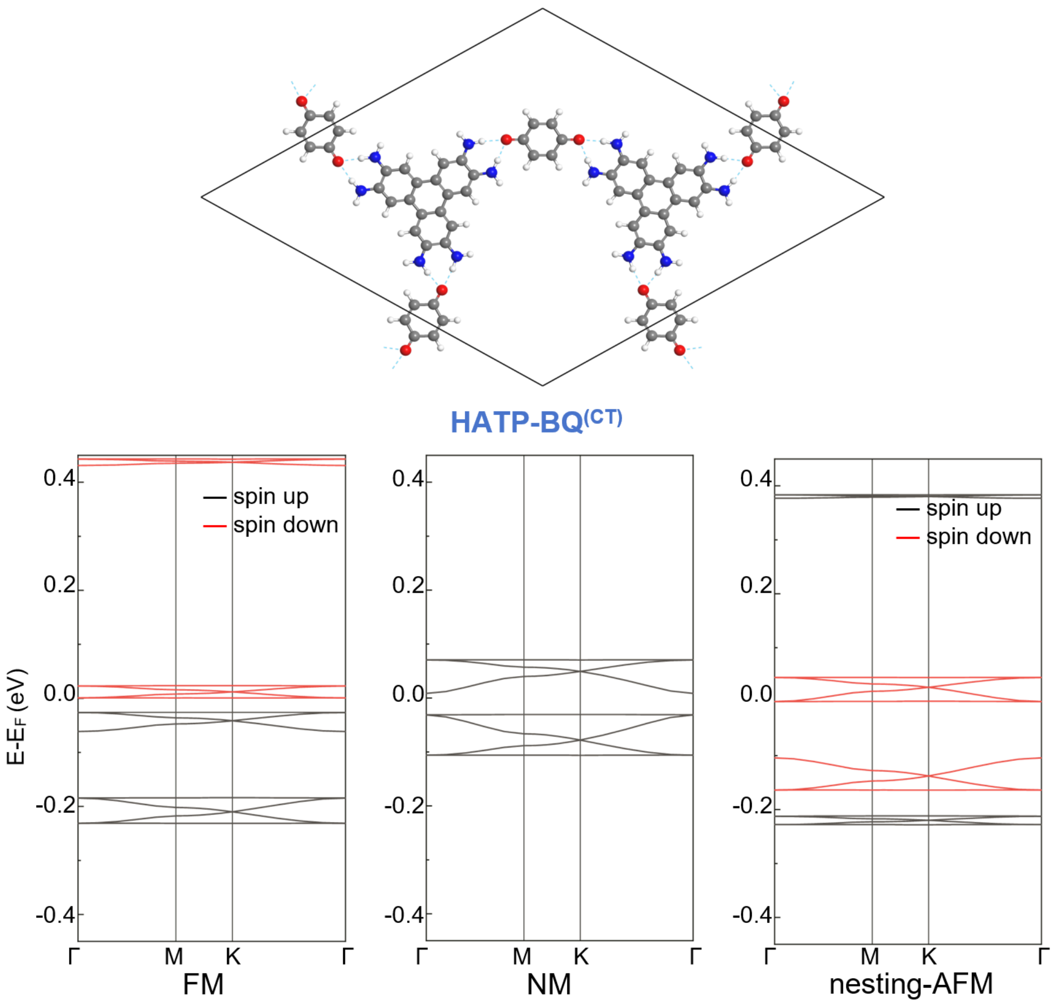

Figure S6. Atomic configuration and band structure of HATP-BQ$^{(CT)}$ HOF under different magnetic states.

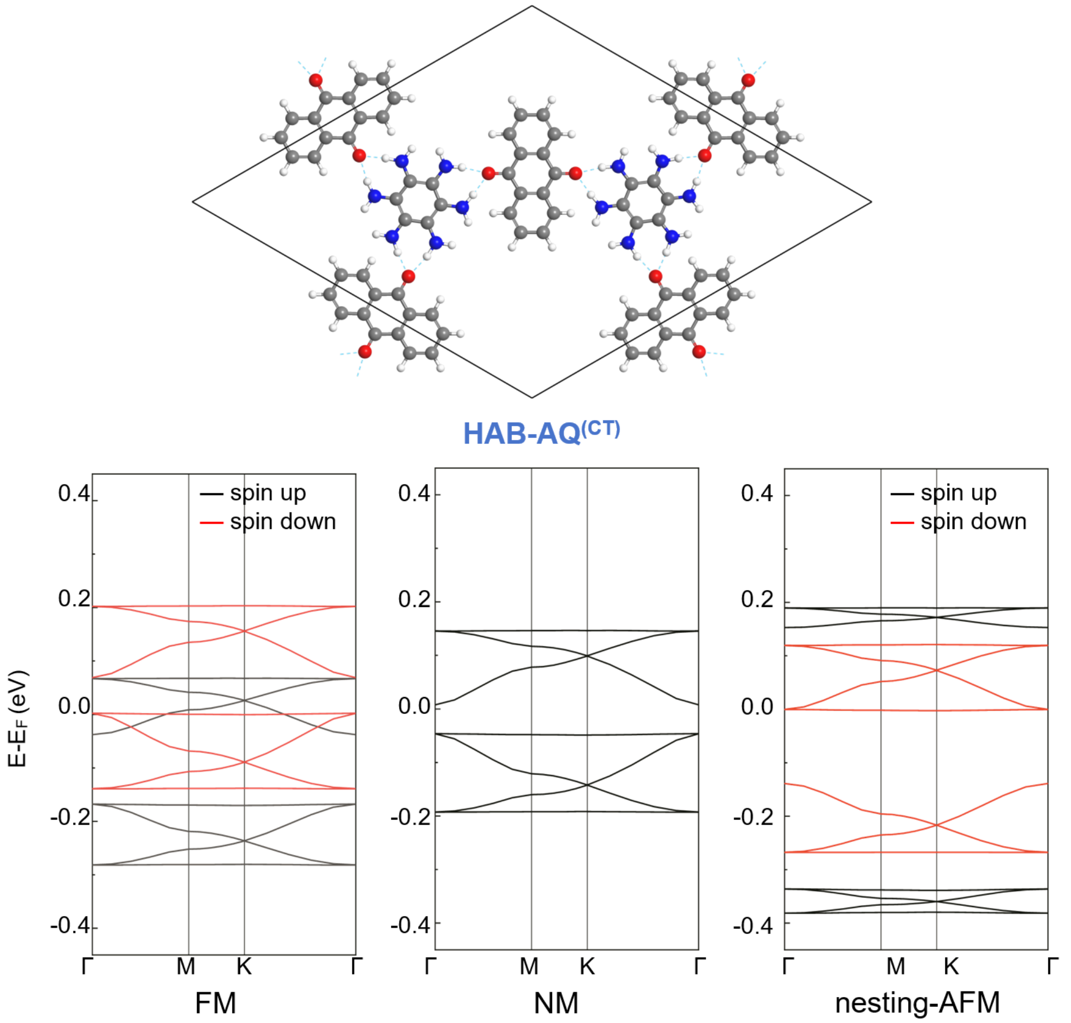

Figure S7. Atomic configuration and band structure of HAB-AQ$^{(CT)}$ HOF under different magnetic states.

2. Schematics of colinear AFM configurations considered in this work.

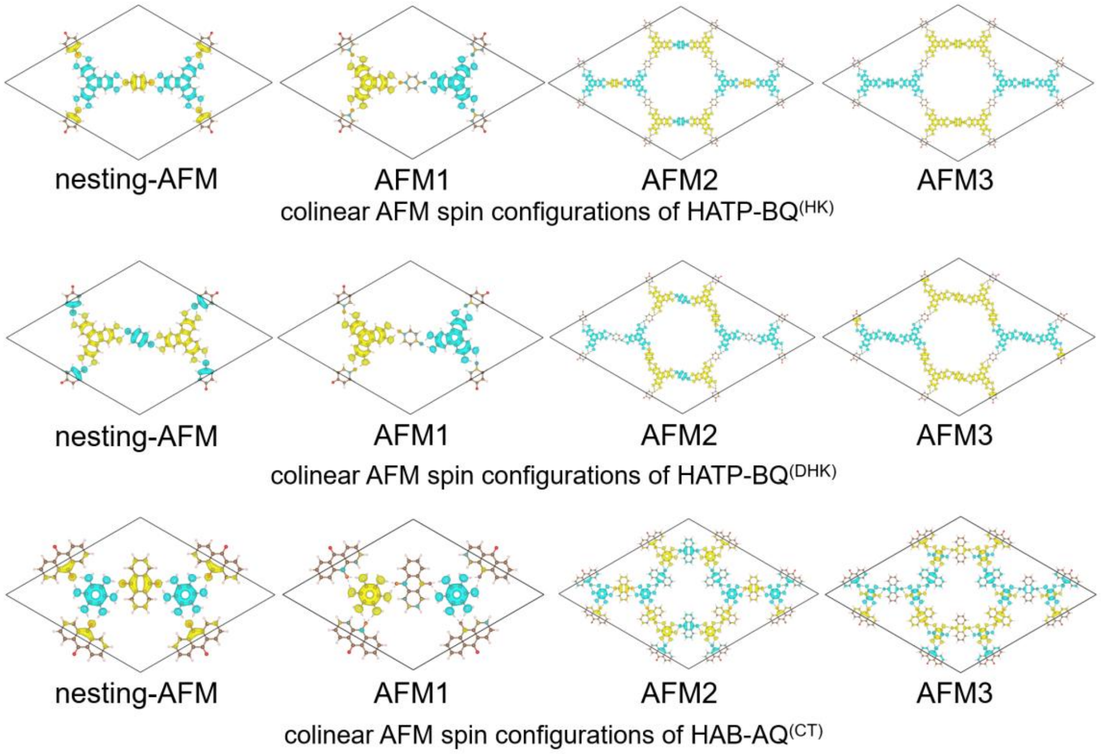

Figure S8. Colinear AFM configurations considered in this work, with HATP-BQ$^{(HK)}$, HATP-BQ$^{(DHK)}$ and HAB-AQ$^{(CT)}$ as representatives.

3. Energy of different magnetic states of the seven HOFs

Table S1. Energy of different magnetic states of the seven HOFs. Energy of the ground state for each HOF is set to zero. Marker "\" means the corresponding magnetic state is unstable.

Magnetism / Energy(meV/uc)	FM	NM	Nesting-AFM	AFM1	AFM2	AFM3	FiM
HAB-BQ$^{(HK)}$	\	147	\	92	55	\	0
HAB-BQ$^{(DHK)}$	0	309	\	\	136	\	3
HAB-BQ$^{(CT)}$	42	417	0	445	178	163	\
HATP-BQ$^{(HK)}$	112	116	0	114	67	111	\
HATP-BQ$^{(DHK)}$	78	254	0	225	152	101	\
HATP-BQ$^{(CT)}$	105	230	0	213	200	142	\
HAB-AQ$^{(CT)}$	119	84	0	67	36	77	\

4. Demonstration of the non-colinear magnetic configurations.

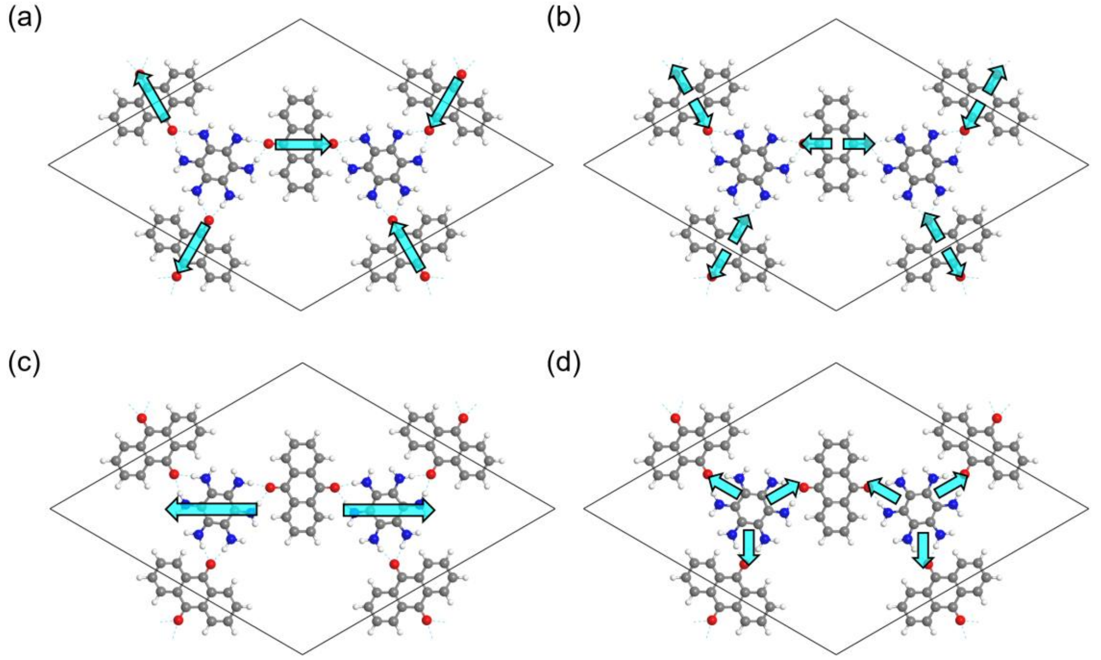

Figure S9. Schematic demonstration of the non-colinear magnetic configurations considered and verified on HAB-AQ$^{(CT)}$ lattice. The arrows represent the in-plane spin polarization directions considered. The calculated results show that the non-colinear configurations exhibit higher energy than the colinear nesting-AFM states. The magnetic configurations exhibit energy that are 110 meV (panel (a)), 129 meV (panel (b)), 70 meV (panel (c), unstable and converge into FM state) and 151 meV (panel (d)) higher than the out-of-plane nesting-AFM state.

5. Verification of the electronic properties with DFT-SCAN functional.

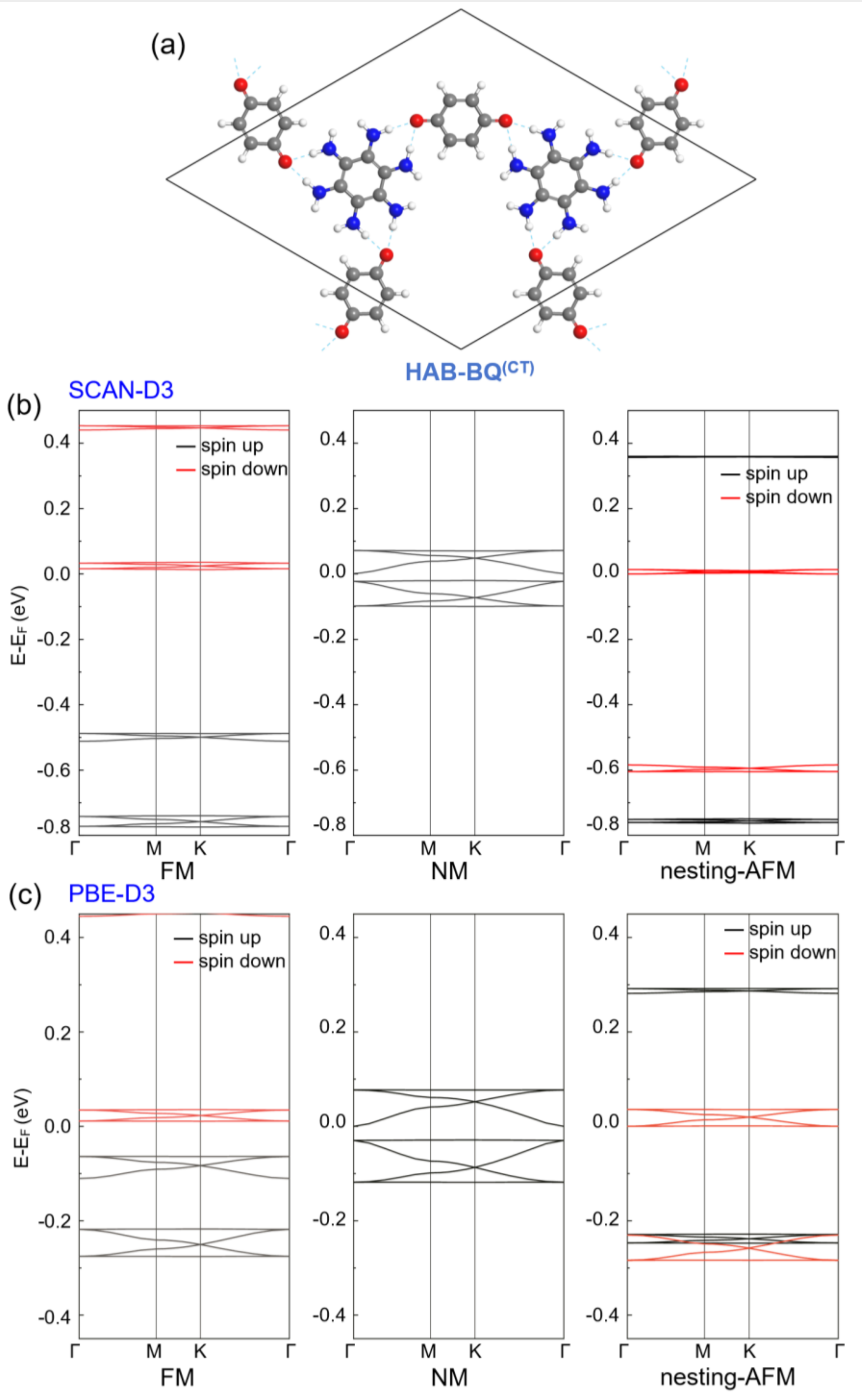

Figure S10. Electronic band structure of HAB-Bq$^{(CT)}$ calculated with DFT-SCAN METAGGA functional (b) compared with PBE calculated band structure (c). Both calculation methods are performed with vdw-D3 corrections. DFT-SCAN functional gives band structures quantitively the same with PBE results, such as the half-metallic nature of nesting-AFM bands, but with significantly smaller band widths and larger gaps between kagome three-band pattern and honeycomb four-band patterns. DFT-SCAN also presents quantitively the same results in terms of magnetic ground states, where the nesting-AFM state remains ground state, 20 meV lower than the FM state and 980 meV lower than the NM state.

6. Comparison of different acceptor molecule choices, with DFB molecule as representative.

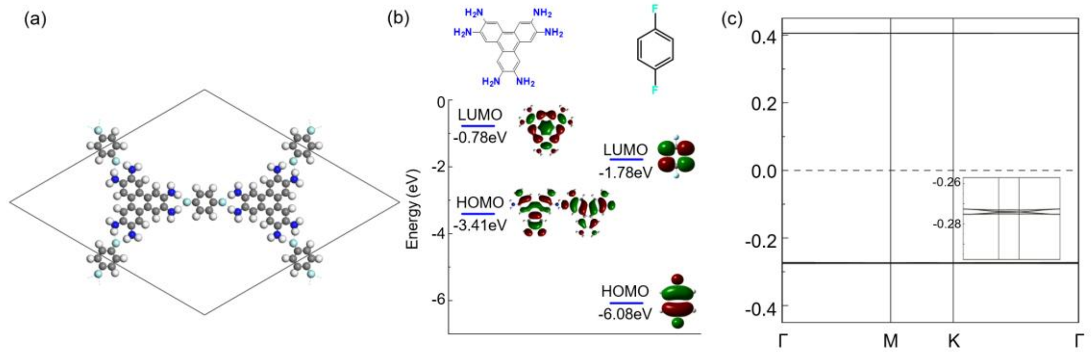

Figure S11. Structure, molecule front orbitals and electronic band structures of HATP-DFB$^{(HK)}$ HOF. Bader charge analysis shows a 0.17 electron charge transfer between HATP sublattice and DFB sublattice, significantly weaker than charge transfer between HATP and BQ in HATP-BQ$^{(DHK)}$ HOF. Judging from molecular front orbitals(b), LUMO of DFB is significantly higher than HOMO of HATP, which leads to significantly weaker charge transfer. This leads to a NM ground state. Due to the weak interactions between molecules, the topological bands show a tiny band width of 5meV, as shown in panel (c).

7. Larger snapshot (25×30 supercell) of the Monte Carlo simulation at 8K.

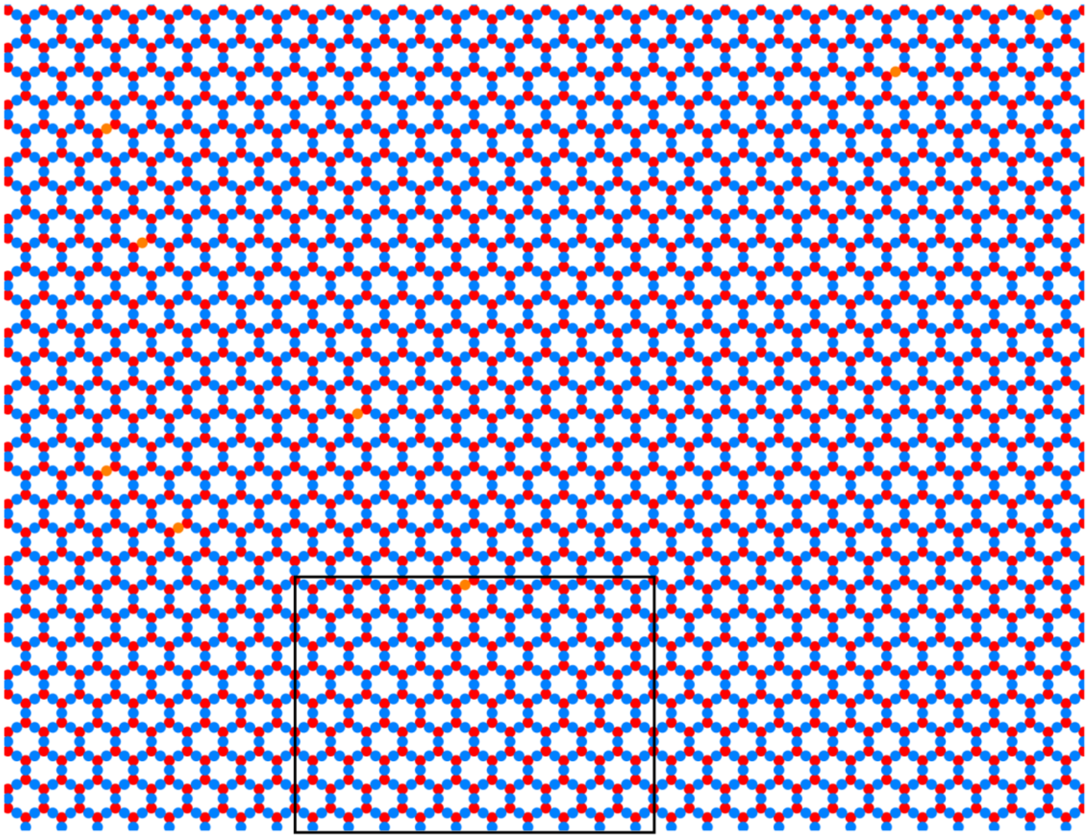

Figure S12. Larger snapshot (25×30 supercell) of the Monte Carlo simulation at 8K. Several kagome sites show different spin polarization directions (the orange dots) due to the limitation of the simulation accuracy. The area presented in Figure 4(b) is marked with black line.

8. Dynamic stability analysis with HAB-BQ$^{(HK)}$ as representative.

To assess the stability of the HAB-BQ$^{(HK)}$ lattice, phonon dispersion calculations were performed. The result is shown in Figure S13, confirming that the freestanding HAB-BQ$^{(HK)}$ structure is dynamically unstable. This instability can be attributed to the presence of twofold-symmetric ligands, such as the acceptor molecules BQ and AQ considered in this work, which are capable of rotating about their central axis. Such rotational freedom leads to dynamic instability in the phonon spectra of the freestanding framework. However, when supported on a substrate, the material can be stabilized through interfacial constraints. A representative example is the Cu-DCA metal-organic framework, which exhibits similar characteristics and has been successfully grown on various substrates [Chem. Commun. 2014, 50, 12289-12292; Nano Lett. 2016, 16, 2072-2075]. Given that materials with unstable phonon dispersions can still be experimentally viable when appropriately supported, practical applicability was evaluated through molecular dynamics simulations on selected substrates, rather than relying solely on phonon analysis. Therefore, calculations with

HOFs on substrates are performed, that are presented in the following Figure S14-S15, which shows that the HOFs could be dynamically stable on appropriate substrates.

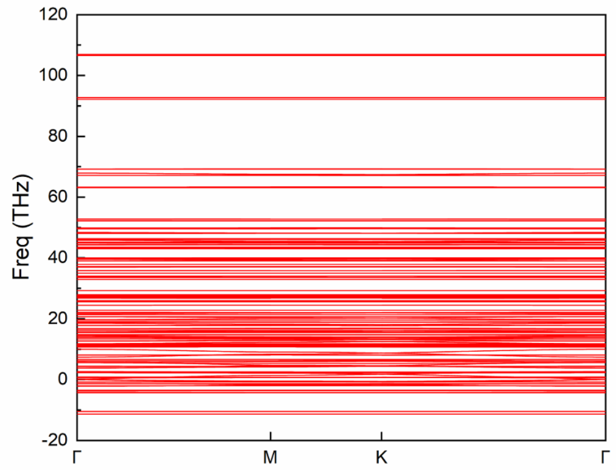

Figure S13. Phonon dispersion of HAB-BQ$^{(HK)}$. Significant optical modes with imaginary frequencies can be observed.

9. Atomic configuration, band structure and molecular dynamic simulation results of HATP-BQ$^{(CT)}$ on WSe_2.

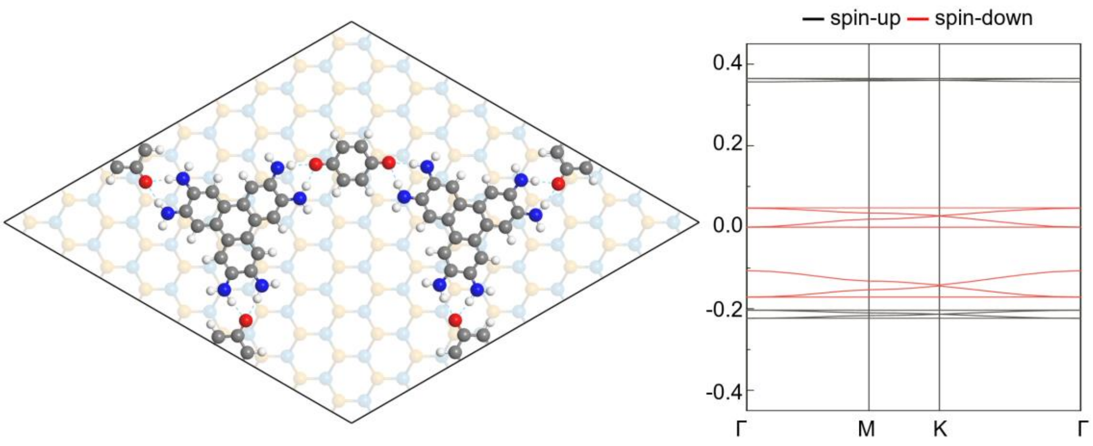

Figure S14. Atomic configuration and band structure of HATP-BQ$^{(CT)}$ on WSe_2. Band structure and magnetic properties of nesting-AFM HATP-BQ$^{(CT)}$ are well preserved.

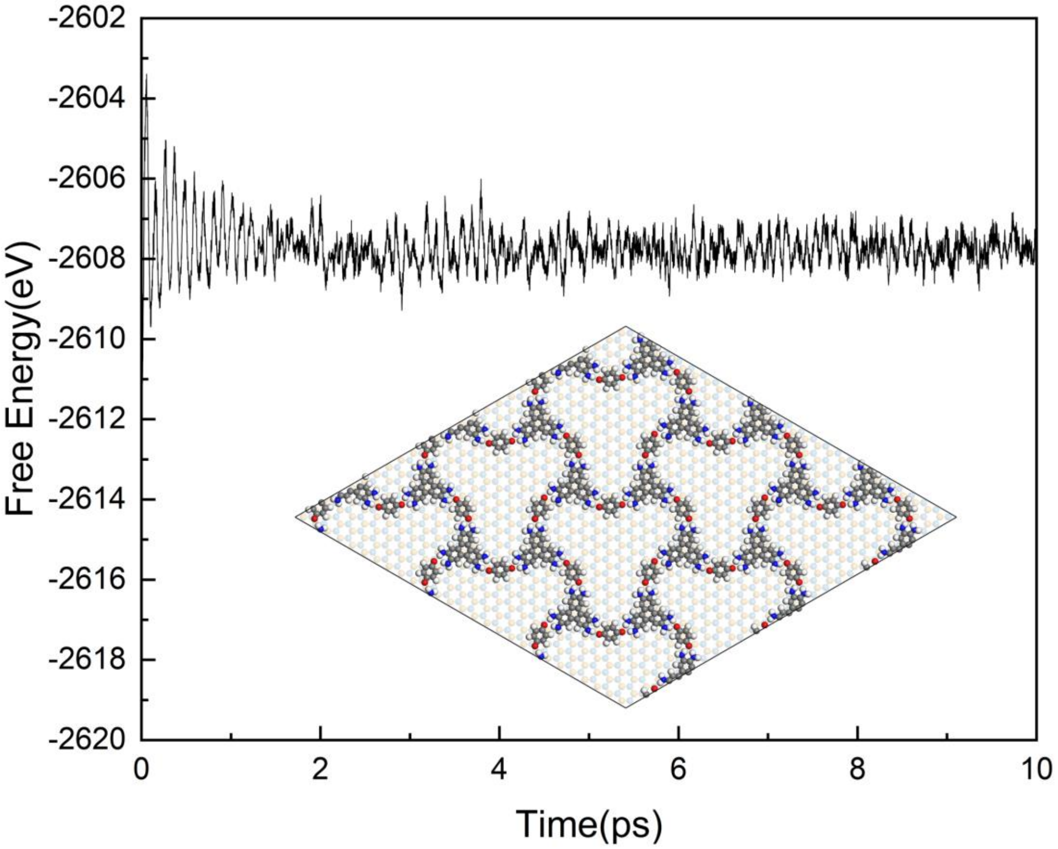

Figure S15. Free energy and structure snapshot for the molecular dynamic simulation of HATP-BQ$^{(CT)}$ on WSe_2 substrate. The HOF structure remains stable on WSe_2 after 10 ps simulation under room temperature.
